# Preoperative, Intraoperative and Postoperative Corticosteroid Use as an Adjunctive Treatment for Rhegmatogenous Retinal Detachment

**DOI:** 10.3390/jcm9051556

**Published:** 2020-05-21

**Authors:** Vincenza Bonfiglio, Michele Reibaldi, Iacopo Macchi, Matteo Fallico, Corrado Pizzo, Clara Patane, Andrea Russo, Antonio Longo, Alessandra Pizzo, Giovanni Cillino, Salvatore Cillino, Maria Vadalà, Michele Rinaldi, Robert Rejdak, Katarzyna Nowomiejska, Mario Damiano Toro, Teresio Avitabile, Elina Ortisi

**Affiliations:** 1 Department of Experimental Biomedicine and Clinical Neuroscience, Ophthalmology Section, University of Palermo, 90127 Palermo, Italy; enzabonfiglio@gmail.com (V.B.); clarapatane@hotmail.it (C.P.); giovannicillino@libero.it (G.C.); salvatore.cillino@unipa.it (S.C.); maria.vadala@unipa.it (M.V.); 2 Department of Ophthalmology, University of Catania, 95100 Catania, Italy; iacopomacchi@gmail.com (I.M.); matteofallico@hotmail.com (M.F.); corradopizzo@hotmail.it (C.P.); andrearusso2000@hotmail.com (A.R.); antlongo@unict.it (A.L.); alepizzo@hotmail.it (A.P.); t.avitabile@unict.it (T.A.); elinaortisi@gmail.com (E.O.); 3 Department of Surgical Sciences, Eye Clinic Section, University of Turin, 10122 Turin, Italy; 4 Department of Ophthalmology, Second University of Naples, 80131 Naples, Italy; michele.rinaldi@unina2.it; 5 Department of General Ophthalmology, Medical University of Lublin, 20079 Lublin, Poland; robertrejdak@yahoo.com (R.R.); katarzyna.nowomiejska@umlub.pl (K.N.); toro.mario@email.it (M.D.T.); 6 Faculty of Medical Sciences, Collegium Medicum Cardinal Stefan Wyszyński University, 01815 Warsaw, Poland

**Keywords:** rhegmatogenous retinal detachment, vitrectomy, scleral buckling, dexamethasone, triamcinolone, fluocinolone, corticosteroids

## Abstract

The treatment for rhegmatogenous retinal detachment (RRD) is surgery, including pars plana vitrectomy (PPV) and scleral buckling (SB). Despite surgical advances, degeneration of the photoreceptors and post-operative complications, such as proliferative vitreoretinopathy (PVR), often occurs as the result of inflammation, preventing complete visual recovery or causing RRD recurrence. There is increasing evidence that in the presence of RRD, the activation of inflammatory processes occurs and the surgery itself induces an inflammatory response. This comprehensive review focuses on the use of different formulations of corticosteroids (CCS), as an adjunctive treatment to surgery, either PPV or SB, for RRD repair. The purpose was to review the efficacy and safety of CCS in improving functional and anatomical outcomes and in preventing postoperative complications. This review is organized according to the timing of CCS administration: preoperative, intraoperative, and postoperative. The evidence reviewed supported the role of the pre-operative use of CCS in the treatment of combined RRD and choroidal detachment (CD), reducing CD height. No solid consensus exists on intraoperative and postoperative use of CCS to treat and prevent postoperative complications. However, a large randomized clinical trial including more than 200 eyes suggested that oral prednisone after surgery decreases the rate of postoperative grade B PVR.

## 1. Introduction

Rhegmatogenous retinal detachment (RRD) is a common retinal disease with an incidence of one in 10,000 people per year [1] that often causes visual field defects and moderate to severe visual impairment. Surgery is the only therapeutic approach and the surgical techniques and instrumentation developed over the last decades have led to a very high primary reattachment rate, which is around 95%, according to various studies [2]. However, despite primary anatomic success, degeneration of the photoreceptors and post-operative complications, such as proliferative vitreoretinopathy (PVR) often occur as a result of inflammation, preventing complete functional recovery or causing RRD recurrence [3,4].

There is increasing evidence that in the presence of RRD, the activation of inflammatory processes occurs, in particular when it is associated with choroidal detachment [5,6]. The surgeries themselves, both scleral buckle (SB) and pars plana vitrectomy (PPV), induces an inflammatory response as demonstrated by an increase of aqueous flare values, from day one up to three months post-operatively with the peak value observed at post-operative day seven [7].

Corticosteroid (CCS) drugs are able to modulate inflammation by binding intracellular receptors and regulating cytokine synthesis [8]. Recently, the use of CCS in addition to surgery, either SB or PPV, has been introduced in the management of RRD to control inflammation, improving reattachment rates and visual recovery, and reducing the incidence of PVR [9]. Several studies have already investigated the efficacy of CCS prior, during, and after surgery for RRD repair, administered in different formulations: topical, subconjunctival, subtenon (ST), intravitreal (IVT) and systemic; however, there is no agreement about the most efficient formulation with the least side effects.

Ando et al. [10] in an experimental study, observed, after injection of 1 mg of intravitreal triamcinolone (TA), a reduction of both blood–retinal barrier (BRB) breakdown and incidence of tractional retinal detachment due to PVR development. Moreover, Bali et al. [11], in a prospective randomized clinical study, reported that preoperative subconjunctival injection of dexamethasone (DEX) significantly decreased postoperative laser flare in RRD eyes treated by SB.

Weijtens et al. [12,13,14,15] reported that the concentration of DEX disodium phosphate in both subretinal fluid and the vitreous of eyes affected by RRD was higher after a subconjunctival injection compared to a peribulbar injection or an oral administration [15]. Shen et al. [16], in a clinical study, and Kovacs at al. [17], in a theoretical model, showed an efficient delivery of Triamcinolone (TA) after an ST injection, measured either in the vitreous chamber or in the choroidal extracellular matrix, despite low serum levels of TA, which seemed to not alter patients’ metabolic balance.

However, other authors [18] observed a significantly higher vitreous concentration of TA after IVT than ST injection, beyond a greater effect on the reduction of the BRB breakdown.

Moreover, CCS use could cause systemic and ocular adverse events (AEs). In particular, systemic AEs include increased risk of serious infections, osteoporosis, Cushing’s syndrome, and insomnia strictly related to high dosage (linear dose-related pattern), depression and increased blood pressure (threshold dose-related pattern, with a high rate of AEs beyond a dose of 7.5 mg/day) [19]. A higher glucose level is another side effect of particular concern, especially in diabetic patients [20]. Ocular complications of corticosteroid use have been well reported after intravenous, oral, topical (ocular and cutaneous), and injected (sub-conjunctival, subtenon, and periocular) corticosteroids. In particular, oral corticosteroid use can cause glaucoma and cataract development with a threshold dose-related pattern (a mean value dosage of over 7.5 mg/day and 5 mg/day, respectively) [21]. The risk of cataract is reported either after intravitreal TA [22] or subtenon injection of TA [23] (up to 15–20%, and up to 2.1%, respectively). An increase in IOP is reported from 18% to 36% of the general population after topical CCS use, although a potentially damaging rise in IOP affects from 5 to 6% of the general population vs. 46% to 92% of patients with primary open-angle glaucoma [21]. Secondary ocular hypertension was also reported after subtenon and intravitreal TA injection (4.7% and up to 40%, respectively) [22,23,24,25] and DEX implant use (up 13%) [26].

The aim of this study was to systematically review the published literature on the use of different formulations of CCS as a therapeutic option in addition to surgery (prior, during, and after surgery), PPV and/or SB, for RRD repair, to investigate the efficacy and the safety of CCS in improving functional and anatomical outcomes and in preventing postoperative complications.

## 2. Methods

### 2.1. Search Methods for Identification of Studies

Literature from the PubMed search engine was analyzed to find current evidence on CSS use in addition to surgery for the management of RRD with or without choroidal detachment; papers published from 2005 up to December 2019 were analyzed. The search strategy used the following keywords and Mesh terms: “periocular, subconjunctival, subtenon, and intravitreal injection; systemic and topic administrations, preoperative, intraoperative, postoperative use of corticosteroids/dexamethasone/triamcinolone acetonide; fluocinolone acetonide; rhegmatogenous retinal detachment/choroidal detachment; SB; PPV; and inflammation”.

### 2.2. Eligibility Criteria

Inclusion criteria were English language and the use of CSS as a therapeutic option in addition to surgery for the treatment of RRD. Articles in which steroids were used as a dye for vitreous, posterior hyaloid or internal limiting membrane, were excluded, as well as articles concerning the use of CCS in the management of postoperative complications such as cystoid macular edema. The reference lists of the analyzed articles were also considered as a potential source of information.

### 2.3. Data Collection

The present review was structured in three sections based on the timing of CCS use in addition to surgery: preoperative, intraoperative, and postoperative.

The following mean characteristics were analyzed for each article:

(1) Study design: retrospective, prospective, comparative and non-comparative, randomized and non-randomized, single-center and multicenter and case report
(2) Clinical outcomes: anatomical and functional
(3) 3Number of eyes studied
(4) Primary treatment
(5) Follow-up (duration of the study)
(6) Main results
(7) Side effects

Two investigators (VB and EO) independently assessed the articles for compliance with the inclusion criteria concerning the selection of the papers to be analyzed and resolved disagreements through consensus.

## 3. Results

**Figure 1:**
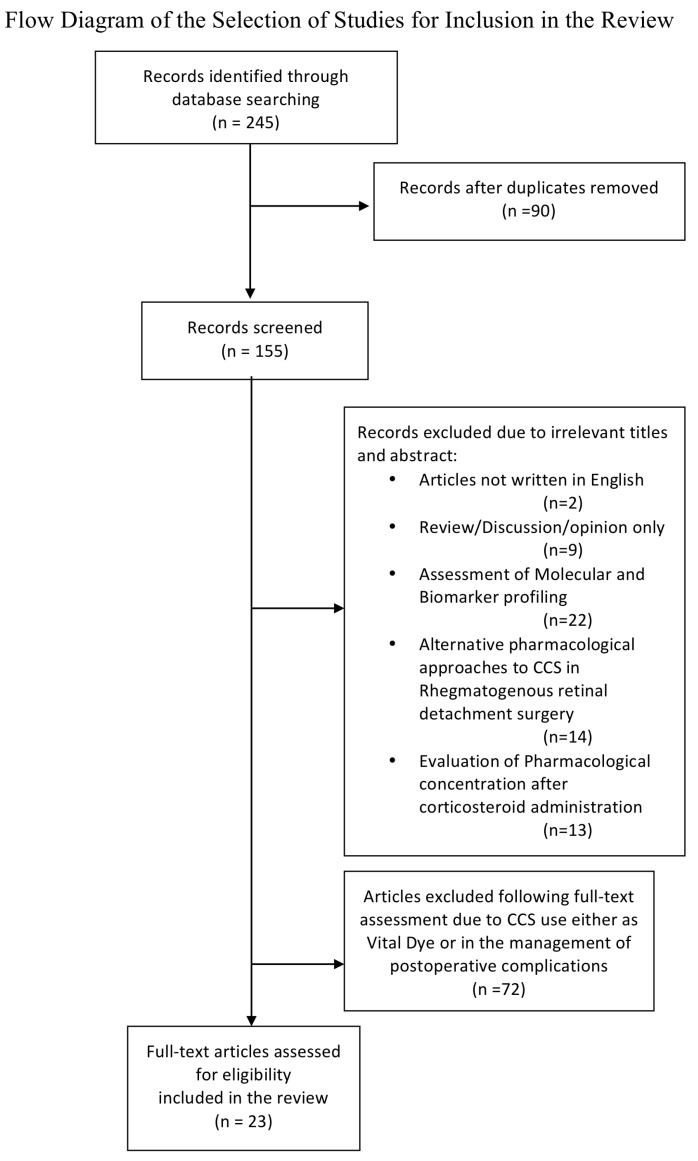
Study selection process.

A total of 245 articles were identified through database searching. After the exclusion of studies on the basis of irrelevant titles and abstracts (such as articles not written in English; studies on assessment of molecular or biomarker profiling, and on the estimation of body and ocular pharmacological concentration of CCS after their administration; evaluation of alternative pharmacological approaches to CCS in RRD surgery) or failure to meet inclusion criteria, 23 studies were assessed as eligible and included in our review (Figure 1).

### 3.1. Preoperative Use

Six studies regarding the preoperative use of CCS were identified by our literature review. Their main characteristics and results are shown in Table 1. Four had a prospective design but only two of these were randomized; the other three of the seven studies were retrospective [27,28,29,30,31,32]. Our systematic review found that CCS are widely used preoperatively in addition to surgery to treat patients affected by RRD associated with choroidal detachment (CD) [27,28,29,30,31,32]. No articles concerning the use of preoperative CCS in RRD without CD surgery were found. Associations of retinal and choroidal detachment (RRDCD) were reported in 2%–4.5% of cases [33]. RRDCD was first, described in 1974 and is characterized by the detachment of the choroid and, often, of the ciliary body too [33].

RRDCD is an uncommon type of retinal detachment, characterized by rapid progression, poor functional and anatomic prognosis due to the more difficult visualization of the ocular fundus and retinal breaks as well as the high rate of postoperative PVR development. [34,35,36] The reattachment rate of RRDCD treated by SB (35.5%–52.4%) was lower compared to that of RRD without CD, according to Seelenfreund et al. [27] After the introduction of primary PPV, the reattachment rate of RRDCD improved by up to 90% [34,35,36].

Risk factors for the development of choroidal detachment are aphakia, pseudophakia, age > 50 years, low IOP, multiple and/or giant retinal breaks, especially when located posteriorly, high myopia, total retinal detachment, and macular hole [37].

The pathogenesis of RRDCD is unclear: vascular and inflammatory processes have been hypothesized. Regarding the vascular pathogenesis, the hypotony induced by the RD has been assumed to lead to CD by stimulating dilatation and hyper-permeability of choroidal blood vessels [38]. In addition, edema of the ciliary body itself could further reduce the production of aqueous humor with a positive feedback loop and consequently more hypotony.

Other authors [6] have shown inflammatory processes playing an important role in the development of CD associated with RRD and hypothesized that a severe uveitic process, secondary to RD, could occur and lead to the exudation of choroidal blood vessels, leakage of fluid and subsequent choroidal detachment with secondary hypotony, creating a vicious circle. This theory found support in the overexpression of inflammatory cytokines and proteins such as migration inhibitor factor (MIF) and soluble intercellular adhesion molecule1 (sICAM-1) observed by Dai et al. [39] in RRDCD eyes, compared with those affected by RRD without CD.

According to these pathogenic theories, the pre-operative use of CCS could play an important role in preparing patients affected by RRDCD for surgery, in order to increase IOP and reduce CD by reducing the permeability of choroidal blood vessels, inhibiting inflammatory reactions and cellular proliferation, and stabilizing BRB [27,28,29,30,31,32].

### 3.2. Intraoperative Use

From our systematic review of the literature, we identified 11 studies [40,41,42,43,44,45,46,47,48,49,50] investigating the effects of steroids as an intraoperative adjuvant in RRD surgery. Of the eleven studies, seven had a prospective design but only five were randomized; three studies were retrospective and one study was a case report.

Overall, in all of these studies, the main reason for the intraoperative use of CCS in combination with PPV was either the treatment of PVR, [41,42,44,48] when associated with RRD, or its prevention, in cases of RRD with a high risk of PVR development [45], and the prevention of postoperative complications such as macular pucker [43], persistent subretinal fluid (SRF) [46,48] and cystoid macular edema [46,48]. PVR is the main cause of surgery failure for RRD repair and its incidence is between 5-11% [51]. It can occur in cases of untreated RRD as well as after any retinal surgical procedure such as laser, criopexy, SB, and PPV [51,52,53]. Although the pathogenesis of PVR is complex and still remains partially unclear, the role of inflammation in the pathogenesis of PVR has been widely accepted and three overlapping biological processes have been identified as major triggers. These are: 1) Retinal pigment epithelial (RPE) and glial cellular migration (into the vitreal cavity and onto the retinal surface, respectively); 2) cellular proliferation (extravasations of blood components such as fibrin, elastin, fibronectin, growth factors, and cytokines secondary to blood–retinal barrier breakdown); and 3) cellular contraction (due to the deposition of the extracellular matrix and collagen synthesis). The final result is the formation of fibrocellular membranes located on the retinal surface and/or on the posterior hyaloid [54,55].

During RRD, the exposure of RPE cells to inflammatory cytokines and growth factors produces several effects. Following retinal detachment, RPE cells lose their polarity and undergo an epithelial-mesenchymal transformation stimulated by transforming growth factor beta (TGF- β). Platelet-derived growth factor (PDGF) stimulates proliferation and is also a chemoattractant for glial cells. Connective tissue factor (CTS-F) promotes cellular migration and proliferation and stimulates the production of extracellular matrix with the consequent formation of membranes. Blood retinal barrier breakdown causes serum growth factor and cytokines to be released into the vitreous, including vascular endothelial growth factor (VEGF), epidermal growth factor (EGF), pigment epithelium-derived factor (PEDF), Platelet-Derived Growth Factor (PDGF), transforming growth factor β (TGFβ), tumor necrosis factor α (TNFα), Fibroblast growth factors (FGF), basic fibroblast growth factor (bFGF), insulin, insulin-like growth factor-1 (IGF-1), interleukin 1 (IL-1), IL-6, IL-8, IL-10, interferon γ (IFNγ), monocyte chemotactic protein, macrophage-colony stimulating factor, granulocyte colony-stimulating factor (G- CSF), chemokine ligand 2 (CCL2), CCL3, CCL4, CCL5, and protein [54,55]. All these factors stimulate cellular migration, cellular proliferation, deposition of cellular matrix, and creation of contractile membranes (growth factor and cytokines hypothesis) [55].

The role of macrophages has been recently highlighted. They have a multifactorial action that includes the secretion of growth factors such as PDGF and their differentiation into fibroblast-like cells (macrophage hypothesis) [55].

Several drugs have been proposed as an adjunctive treatment for preventing postoperative PVR. These include 5-Fluorouracil (5-FU) Low-Molecular-Weight Heparin (LMWH), Daunorubicin, 13-Cis-Retinoic Acid, Cyclin-Dependent Kinases, and Corticosteroids [55].

Corticosteroid treatment can modulate both the inflammatory and proliferative pathways of PVR by stabilizing the blood–retinal barrier and suppressing local growth factors and inflammatory cytokines. This could finally lead to the inhibition of the proliferation of RPE cells, fibroblasts, and myofibroblasts, [55,56].

TA, DEX, and fluocinolone acetonide (FA) implants are the only three CCS approved treatments for intravitreal use. These molecules differ from each other based on their pharmacokinetic and pharmacodynamic properties, with consequently different biological effectiveness, such as different glucocorticoid binding affinity (DEX > FA > TA) and different anti-inflammatory activities (DEX = FA and 5 times more active than TA) [57]. The advantage of a DEX implant is the slow release of active dexamethasone within the vitreous chamber for up to six months, with a single injection, and with a similar clearance in vitrectomized and non-vitrectomized eyes [58]. However, 4 mg TA intravitreal injection has been reported to have an effect that lasts up to three months [59] with a six times quicker clearance in vitrectomized than in non vitrectomized eyes [60].

The FA implant showed similar efficacy in the treatment of chronic diabetic macular edema in both vitrectomized and not vitrectomized eyes [61]. It has also been reported to have a longer action compared to the DEX implant in vitrectomized eyes [62]. Among the 11 studies reviewed, we identified 6 studies investigating the use of intravitreal triamcinolone (IVTA) and 3 studies assessing the use of the DEX implant in addition to surgery to treat RRD. Moreover, we found one study that investigated the use of intravenous dexamethasone during surgery. No studies regarding the intraoperative use of the FA implant were found. The main characteristics and results of these studies are shown in Table 2.

Previous experimental and clinical research demonstrated that IVTA was not significantly retinotoxic when used during vitrectomy without silicone oil (SO) endotamponade [63,64,65,66]. On the other hand, toxicity is still controversial when IVTA (in different doses: 2, 4, 10, and 20 mg) is used during vitrectomy with SO endotamponade [67,68,69]. Kivilcim et al. [67] reported no toxic effects when TA was injected into silicone-filled eyes with a dose up to 5 mg. However, Perkins et al. [68] and Jonas J B [69] suggested that SO might increase TA absorption, prolonging its permanence in the vitreous cavity. Moreover, Spritzer [70], in an experimental model, observed that TA injected into the vitreous cavity filled with SO precipitated at the lower border of the endotamponade bubble, without mixing with it and causing a possible cytotoxic effect on the retina.

In conclusion, it is still unclear which is the best timing for TA injection: soon before silicone oil injection [40] or at the end of the operation into the silicone oil bubble filling the vitreous chamber [41].

Fewer data exist about a DEX implant into silicone oil-filled eyes. One author, using an in vitro model, showed that when DEX is injected into vitrectomized silicone oil-filled eyes, SO modified and increased DEX release over a one-year period [71]. To date, in vivo information is limited to a few case reports and only one recent randomized clinical trial (RCT), with the latter showing results that a DEX implant is generally well tolerated. On the other hand, Bakri and Alniemi [72] reported epiretinal fibrosis development around the implant at the 6th postoperative week, leading to recurrent retinal detachment that needed vitrectomy, removal of the implant, and peeling of epiretinal proliferation.

### 3.3. Postoperative Use

Five studies assessing the postoperative use of CCS (oral or topical) were identified by our literature review. [7,73,74,75,76] All of them had a prospective design but only two of these were randomized. Their main characteristics and results are shown in Table 3.

The postoperative use of CCS was suggested to modulate postoperative pain and inflammation in order to obtain better compliance in terms of patient positioning and postoperative examinations [7]. Moreover, postoperative and intraoperative CCS use could improve surgical results decreasing postoperative complications including PVR, [73,75] macular pucker, [73,75] macular edema [73] and persistent subretinal fluid [74]. Persistent SRF occurs in 27–100% of cases, even four to six weeks after the operation, and is associated with poor vision [77,78].

Several factors have been postulated to contribute to the pathogenesis of persistent SRF, such as the height and extent of retinal detachment, clock hours of buckle placement, gender, age, refractive status, and SRF drainage. However, little evidence of any association has been shown between these factors and the presence of persistent SRF, except for the extent of retinal detachment [79]. According to previous reports, persistent macular SRF often lasts a long time before it is completely reabsorbed. Median duration of persistent SRF ranges from 5 to 10 months, and most SRF disappears within one year after surgery [79,80]. Previous studies found that the incidence of residual SRF was reduced by anti-inflammatory agents such as steroid and traditional non-steroid anti-inflammatory drugs showing that inflammation could play a part in the pathogenesis of persistent SRF. These drugs reduce the blood–retinal barrier breakdown that might be one of the possible mechanisms of persistent SRF after retinal detachment surgery [74,80].

## 4. Conclusions

In this systematic review, we examined the use of CCS in different formulations (topical, subconjunctival, ST, IVT, and systemic) in addition to surgery for RRD treatment using either SB or PPV.

The evidence suggested that preoperative CCS use could play a role only when RRD is associated with choroidal detachment thanks to its efficacy in decreasing the maximum CD height [29,30,31] and in reducing the complexity of surgery and potential complications related to CD. Dewattana et al. [31] reported a highly significant preoperative improvement (partial or complete) of CD in eyes treated with preoperative CCS (oral prednisolone or subtenon injection of triamcinolone), for a median of 7 days before surgery, compared with eyes that did not receive any preoperative CCS treatment. (30% vs. 82%; *p* > 0.001). However, preoperative CCS use did not provide better postoperative visual and anatomical outcomes [27,28,29,31].

On the contrary, Sharma et al. [27], in their prospective randomized study, reported better functional results in eyes treated immediately by PPV without CCS administration with respect to a group treated with oral prednisolone (1 mg/kg body weight) for 7 days prior to PPV, (improvement of visual acuity of two lines or better was 89% vs. 73%, respectively). The authors reported that preoperative CCS use could delay surgery (7.5 days vs. 3 days, in the steroid-group and no-steroid group, respectively; *p* < 0.001), increasing the risk of photoreceptor degeneration and apoptosis. Likewise, persistence of CD in 2 of 11 eyes was reported [27], despite preoperative CCS use, and no differences in terms of postoperative complications, including PVR, between the two groups were registered.

Taking all this into account, preoperative CCS administration was considered to promote preoperative resolution of CD, a single subtenon injection of TA [29] was more effective in CD improvement than endovenous CCS administration, with the advantage of inducing significantly lower blood sugar levels and lower suppression of plasmatic cortisol levels [29].

The most common reported adverse event after either preoperative periocular or subtenon injection of TA [29] was an elevated IOP that, however, was successfully treated by IOP-lowering medication, with no glaucoma surgery required in most cases. Cataract progression was also reported by Wei Y et al. [28] after both oral prednisolone administration (16.1%) and 40 mg methyl-prednisolone periocular injection (21.7%), given 3 to 7 days before surgery, however, it was very difficult to establish the role of vitrectomy, silicone oil tamponade and CCS in cataract progression.

Intraoperative intravitreal use of TA and DEX implant in addition to PPV were also investigated. In the literature, the aim of their use in eyes affected by RRD, was either the treatment of PVR itself [41,42,44,48] or the prevention of postoperative complications such as PVR [45], macular pucker, [44] persistent SRF and cystoid macular edema [46,48]. However, the evidence is limited and the results are still inconsistent. Comparative prospective controlled clinical trials [42,43] did not report any advantages in terms of both visual and anatomical outcomes or in prevention of macular pucker development and recurrent PVR rate in eyes treated by PPV and intraoperative IVTA injection [42,43]. In addition, an increased risk of IOP elevation was reported [18,42,43,46].

Mirshahi A et al. [46] in a prospective consecutive comparative study, investigating the effect of IVTA on the resolution of subretinal fluid after SB surgery, observed that a single IVTA injection increased the final BCVA in macula-off RRD patients despite persistent SRF (34% of SRF in IVTA eyes vs. 45% in placebo eyes), suggesting that anti-inflammatory drugs could play a role in the good results of SB surgery.

Banerjee PJ et al. [48], in a prospective randomized clinical trial, including a large sample of 140 eyes treated for RRD by PPV with silicone oil and intraoperative DEX implant, did not observe any benefits compared with the control group at a six-month follow-up, neither in terms of retinal reattachment stability, after SO removal, nor in final VA or PVR recurrence rate and quality of life. However, the authors reported a lower postoperative macular edema rate in the DEX implant group than the control group (42.7% vs. 67.2%) and a higher number of AEs in the control group, suggesting a beneficial effect of additional anti-inflammatory activity of the DEX implant and hypothesized that neural retinal remodeling, secondary to RD, could be the primary cause of the poor postoperative visual outcomes despite less postoperative macular edema. However, there were more episodes of intraocular high pressure in the DEX implant group than the control group, even if there was no difference in terms of diagnosis of glaucoma between the two groups.

Recently, Reibaldi et al. [50] suggested that combined intravenous therapy with dexamethasone and ondansetron, administered intraoperatively, could reduce the incidence and severity of suprachoroidal hemorrhage and intraocular bleeding that are well-known postoperative complications that could worsen anatomical and functional outcomes.

We also reviewed the use of postoperative CCS use in the treatment of RRD. In the literature, it has been reported that the use of systemic postoperative CCS could reduce inflammation and counteract the increased levels of inflammatory cytokines and VEGF associated with a breakdown of the blood–retina barrier responsible for postoperative PVR development.

In clinical practice, the efficacy of the postoperative use of systemic CCS after SB for RRD was tested in two randomized trials reporting controversial findings. Dehghan et al. [73] did not observe any visual or anatomical improvement or reduction in postoperative complications (CME, PVR, and CD) using oral prednisolone 1 mg/kg for 10 days after surgery.

On the contrary, Koener et al. [75] in 220 eyes, reported that oral prednisone at the initial dosage of 100 mg for six days, thereafter tapered to 50 mg for five days, and 12.5 mg for another four days, was effective in reducing the incidence of postoperative complications such as PVR stage B (26.7%, 23.6%, and 19.8% in the steroid group and 41.8%, 46.9%, and 39.1% in the placebo group, respectively, at one, three, and six months postoperatively).

Similarly, Wu et al. [74] reported the effectiveness of oral prednisolone for 3 days after SB, in significantly reducing SRF with respect to control eyes (56.6% vs. 80.0%). Additionally, the authors observed in patients with persistent SRF, a shorter period for fluid reabsorption in the CCS group rather than the control group (218.1 6 122.1 days vs. 286.5 6 141.0 days; *p* = 0.039).

The postoperative use of topical steroids could be used to reduce postoperative pain and flare, however, topical diclofenac sodium seems to be as potent as topical CCS in managing postoperative inflammation, either after PPV or SB, with better analgesic effects and lower intraocular hypertension rata [7,76].

The main limitation of this review was the heterogeneity of clinical variables among the included studies. Reviewed studies could have been widely different with regards to the specific characteristics of the detachment (advanced PVR, state of the macula, trauma, myopia, pseudophakic and recurrent RRD) as well as the patient’s general conditions (including underlying systemic or ocular inflammatory disease, diabetes mellitus). These different clinical characteristics could have had an influence on the baseline inflammatory state and the outcomes specifically related to steroid administration. Therefore, when the findings of the included studies were analyzed all together, the significance of steroid administration for any given primary pathology could have been partially masked by these confounding factors.

## 5. Recommendation

In conclusion, the available published evidence showed that the pre-operative use of CCS has a role only in the presence of combined rhegmatogenous retinal and choroidal detachment: it reduces CD height, even if no anatomical and functional gains were reported. Besides, its use could delay the operation, which, in turn, can lead to photoreceptor loss and reduced visual outcome. The intraoperative use of intravitreal TA or DEX implant either with or without silicone oil mainly aims to prevent postoperative PVR, but the results are still controversial, and no consensus exists for their use. Likewise, no solid consensus exists on the effectiveness of the systemic postoperative use of CCS with the purpose of reducing postoperative complications such as PVR, pucker, cystoid macula edema, and persistent SRF. However, a large RCT including more than 200 eyes suggested that oral prednisone after SB surgery decreased the rate of postoperative PVR grade B. Additionally, postoperative oral prednisolone could reduce the incidence of SRF after SB. Regarding topical postoperative CCS, topical diclofenac seems to be as potent as topical dexamethasone in managing postoperative inflammatory response induced by surgery for RRD with a better analgesic effect and more safety.

## Figures and Tables

**Table 1:** Preoperative use of corticosteroid drugs in retinal detachment surgery.

Author (Year)	Study Design	Outcomes	Number of Eyes	Primary Treatment	Follow-Up	Main Results	Side Effects
Sharma T et al. (2005) [27] India	Prospective randomized study	1. Anatomic primary success 2. Anatomic final outcomes 3. Functional success (≥2 lines improvement)	20 RRDCD eyes: (4 eyes PPV; 16 PPV + SB)	GROUP 1 (11 eyes) oral prednisolone for 7 days before PPV GROUP 2 (9 eyes) early PPV	Group 1 11.7 months (mean) Group 2 mean 30.3 months (mean)	1. Anatomic primary success 81.8% (group 1) vs. 66.7 (group 2) *** 2. Anatomic final success 100% (group 1) vs. 100% (group 2) *** 3. Functional outcomes: ≥ 2 lines improvement 72.73% (group 1) vs. 88.9% (group 2) ***	No specified
Sharma T et al. (2005) [27] India	Prospective randomized study	1. Anatomic primary success 2. Anatomic final outcomes Functional success (≥2 lines improvement)	20 RRDCD eyes (4 PPV; 16 PPV + SB)	GROUP 1 (11 eyes) oral prednisolone for 7 days before PPV GROUP 2 (9 eyes) early PPV	Group 1 11.7 months (mean) Group 2 30.3 months (mean)	1. Anatomic primary success 81.8% (group 1) vs. 66.7% (group 2) *** 2. Anatomic final success 100% (group 1) vs. 100% (group 2) *** 3. Functional outcomes: ≥ 2 lines improvement 72.73% (group 1) vs. 88.9% (group 2) ***	No specified
Wei Y et al. [28] (2014) China	Retrospective clinical trial	1. Retinal reattachment rate after single operation 2. BCVA improvement	77 RRDCD eyes treated by PPV: GROUP A (31 eyes) Oral prednisolone for 3 to 7 days before PPV GROUP B (46 eyes) 40 mg peri-ocular injection on methyl-prednisolone for 3 to 7 days before surgery	GROUP A1 (18 eyes) PPV + SO GROUP A2 (13 eyes) PPV + C3F8 GROUP B1 (17 eyes) PPV + SO + IVT TA (4 mg) GROUP B2 (15 eyes) PPV + SO GROUP B3 (14 eyes) PPV + C3F8	12 months	1. Retinal reattachment rate: • 77.4% (group A) vs. 73.9% (group B) *** • 83.3% (group A1) vs. 82.4% (group B1) *** • 69.2% (group A2) vs. 83.3% (group A1) *** • 73.3% (group B2) vs. 82.4% (group B1) *** • 64.3% (group B3) vs. 82.4 (group B1) *** 2. BCVA improvement: • 83.9% (group A) vs. 80.4% (group B) *** • 88.9% (group A1) vs. 88.2% (group B1) *** • 76.9% (group A2) vs. 88.9% (group A1) *** • 80.0% (group B2) vs. 88.2% (group B1) *** • 71.4% (group B3) vs. 88.2 (group B1) ***	Cataract development: • Group A = 5/31 yes • Group B = 10/46 Glaucoma: • Group A = 5/31 eyes • Group B = 11/46 eyes
Shen LJ et al. [29] (2016) China	Prospective study	1. Pre-operative CD change 2.Blood sugar 3. Systemic and vitreous steroid levels 4. Macular thickness by OCT 5. BCVA change	30 RRDCD eyes treated by PPV	- TA GROUP (16 eyes) ST injection of TA 5 days before surgery - DEX GROUP (14 eyes) 10 mg dexamethasone sodium phosphate EV once a day for 5 days prior to surgery	6 months	TA GROUP vs. DEX GROUP (1) IOP elevation §: 3.29 ± 4.56 mmHg vs. 1.16 ± 1.60 mmHg ** (2) CD height decrease §: 3.55 ± 1.33 mm vs. 1.84 ± 1.5 mm ** (3) Increase of blood sugar level §: 5.75 ± 1.08 mmol vs. 9.01 ± 3.3 mmol/L ** (4) Steroid levels: Aqueous = 85.03 ± 72.92 ng/mL (5 days) vs. 89.57 ± 88.53 ng/mL (45 min) ** Vitreous = 17.95 ± 10.67 ng/mL (5 days) vs. 15.65 ± 10.87 ng/mL (45 min) ** (5) Suppressed plasma cortisol level = 51.9 ± 35.9 ng/mL vs. 8.35 ± 10.35 ng/mL ** (6) Macular thickness: 1 month = (13 eyes) 256 ± 66 vs. (10 eyes) 401 ± 96 ** 3-month = (11 eyes) 260 ± 68 vs. (10 eyes) 319 ±130 *** (7) BCVA change (LogMAR): 1 month = 1.33 ± 0.61 vs. 1.51 ± 0.56 3 month = 1.21 ± 0.59 vs. 1.38 ± 0.58 ***	• IOP elevation (>21 mmHg): - 4 eyes in TA group vs. 1 eye in DEX group • Retinal re-detachment: 1 eye in TA group
Alibet Y et al. [30] (2017) Switzerland, Ukraine	Prospective non-randomized study	1. CBT by UBM 2. Ciliary body and choroidal reattachment 3. Sign on intraocular inflammation	49 RRDCD eyes treated by PPV and pre-operative topical dexamethasone phosphate 0.1 and cyclopentolate hydrochloride	GROUP 1 (30 eyes) received 4 mg of TA IVT GROUP 2 (19 eyes) 4 mg of TA IVT + 0.4–0.8 mL of perfluorpropane	Non specified	1. Total mean CBT value from baseline following IV injection = from 0.83 (0.09) mm to 0.65 (0.09) mm ** 2. 100% ciliary body and choroidal reattachment rate in both groups 3. No sign of intraocular inflammation (ciliary tenderness conjunctival injection and posterior synechiae) 1-2 days after IVTA	IOP increase from 6.9 (1.5) to 13.3 (0.9) mmHg 1–4 days after TA IVT. **
Denwattana A et al. [31] (2017) Thailand	Retrospective study	1. Retinal reattachment rate 2. BCVA improvement at 3 months 3. CD improvement prior to surgery	76 RRDCD eyes treated by PPV or PPV + SB	GROUP A (37 eyes) no pre-operative steroids GROUP B (39 eyes) steroids for a median of 7 days prior to surgery: - 34 eyes: - oral prednisone 0.5–1mg/kg/day - 5 eyes: 20 mg in 2 eyes, 40 mg in 3 eyes of ST TA	20 months (mean)	1. Reattachment rate at 3 months: after 1 operation: 59% (group A) vs. 51% (group B) *** after 2 operation: 70% (group A) vs. 69% (group B) *** 2. BCVA improvement at 3 months: Group A from 2.54 to 2.01 LogMAR ** Group B from 2.53 to 1.97 LogMAR ** Group A vs. group B *** 3. Preoperative CD improvement: None = 70% group A vs. 18% group B ** Partial = 24% group A vs. 46% group B ** Complete = 6% group A vs. 36% group B **	—
Yu Y et al. [32] (2019) China	Retrospective study	1. Retinal reattachment rate 2. Factors influencing primary reattachment rate 3. Factors influencing final reattachment rate	175 RRDCD eyes treated by PPV + preoperative variables	GROUP 1 (59 EYES) no pre-operative steroid treatment GROUP 2 (43 eyes) oral prednisolone 1 mg/kg/day for 5 to 7 days before surgery GROUP 3 (32 eyes) peri-ocular injection of 20 mg methylprednisolone every other day for 5 to 7 days before surgery GROUP 4 (41 eyes) pre-operative 4 mg TA IVT for 5 to 7 days before surgery	—	1. Retinal reattachment rate: - Overall = 72.57% (primary reattachment°) vs. 89.14% (final reattachment) - Group 1 (69.49%) vs. Group 2 (74.42%) ** - Group 4 vs. Group 1 ** (OR = 4.60) 2. Baseline factors influencing primary reattachment rate: - Age = primary reattachment increase with increasing age ** (OR = 1.03). - PVR grade C vs. A-B (OR = 0.31) ** PVR grade D vs. PVR A-B (OR = 0.03) ** - Steroid treatment: Group 1 (69.49%) vs. group 2 (74.42%) *** Group 1 (69.49%) vs. group 3 (62.50%) *** Group 1 (69.49%) vs. group 4 (82.93%) ** 3. Baseline factors significantly influencing final reattachment rate: - Age = final reattachment increase with increasing age (OR = 1.05) **	—

Footnote: § Changes between pre-steroid treatments and pre-vitrectomy procedure; ** statistical significance; *** non statistical significance difference between groups; ° Primary = 1 single operation; AC = Anterior chamber; BCVA = Best corrected visual acuity; C3F8 = Octafluoropropano; CBT = Ciliary body thickness; CD = Choroid detachment; DEX = Dexamethasone; IOP = Intraocular pressure; IV = intravitreal; IVTA = Intravitreal Triamcinolone; EV = Endovenous; OR = Odds ratio; PPV = Pars plana vitrectomy; PVR = Proliferative vitreoretinopathy; RRD = Rhegmatogenous retinal detachment; RRDCD = Rhegmatogenous retinal detachment with combined choroidal detachment; SB = Scleral buckling; SO = Silicone oil; ST = Subtenon; TA = Triamcinolone Acetonide.

**Table 2:** Intraoperative use of corticosteroid drugs in retinal detachment surgery.

Author, Year	Study Design	Outcomes	Number of Eyes	Primary Treatment	Follow-Up	Main Results	Side Effects
Munir WM et al. [40] (2005) United States of America	Retrospective study	1. Visual acuity 2. IOP changes	13 RRD eyes + PDR + severe PVR	PPV + SO + IVTA (4 mg)	Mean = 4.7 months (range 1–15)	1. Visual acuity: - Improvements = 4 eyes (2 lines) - Worsened = 4 eyes - Stable = 5 eyes 2. IOP changes: From 10.8 ± 6.22 mmHg (pre-operative) to 9.6 ± 3.86 mmHg (last follow-up) ***	—
Cheema RA et al. [41] (2007) United Kingdom	Interventional non comparative prospective study	1. Retinal reattachment rate 2. BCVA improvement	24 RRD eyes + PVR grade ≥ C2	PPV + membrane peeling + SO + IVTA (4 mg)	6 months	1. Reattachment rate: 87% 2. BCVA improvement (*p* < 0.5)	—
Admaideh H et al. [42]^36^ (2008) Iran	Prospective randomized clinical trial	1. Retinal reattachment rate 2. BCVA improvement 3. Rate of recurrent PVR 4. Redetachment rate 5. Macular pucker	75 RRD eyes + PVR grade C treated by PPV + SO	GROUP 1 (38 eyes) IVTA GROUP 2 (37 eyes) no IVTA (4 mg)	6 months	1. Retinal reattachment rate:84.2% (group 1) vs. 78.4% (group 2) *** 2. BCVA improvement (logMAR)^: Group 1 from 2.1 ± 0.7 to 1.2 ± 0.7 ** Group 2 from 2.4 ± 0.6 to 1.4 ± 0.6 ** Final BCVA group 1 vs. group 2 *** 3. Rate of recurrent PVR grade C: 28.9% (group 1) vs. 29.7% (group 2) *** 4. Reattachment rate: 15.8% (group 1) vs. 21.6% (group 2) *** 5. Macular pucker: 21.1 (group 1) vs. 35.1 (group 2) ***	Pseudohypopyon = 2 eyes. Rise of IOP^: - Group 1 from 9.5 ± 5.8 vs. 14.7 ± 5.1 mmHg ** - Group 2 from 11.2 ± 7.2 to 16.4 ± 5.9 mmHg ** - Group 1 vs. group 2 ***
Yamakiri K et al. [43] (2008) Japan	Multi-center prospective controlled clinical trial	1. Changes of visual acuity 2. Post-operative complications (ERM, IOP increase) 3. Additional surgery	774 eyes treated by PPV (various disease)	GROUP 1 (391 eyes) TA-assisted PPV (20.5% RRD) GROUP 2 (383 eyes) conventional PPV (20.1% RRD)	1 year	1. Changes of visual acuity: - Improvement Group 1 (322 out of 391 eyes) vs. Group 2 (312 out of 383 eyes) *** - DeteriorationGroup 1 (48 out of 391 eyes) vs. Group 2 (26 out of 383 eyes) *** 2. Post-operative complications: Group 1 vs. Group 2 *** 3. Additional surgery: Group 1 vs. Group 2 ***	—
Chen et al. (2011) [44] China	Retrospective interventional case series	1. Retinal reattachment rate BCVA improvement (≥ 0.3 logMAR)	32 eyes with PVR grade C or D secondary to RRD 5 eyes with PVR grade C or D secondary to ocular trauma	PPV + SO + membrane peeling + IVT TA (2 mg)	Mean = 22.9 ± 9.6 months	1. Reattachment rate = 97.3% 2. BCVA (logMAR): - 1.76 ± 0.56 (baseline) vs. 0.87 ± 0.56 (last follow-up) ** - 1.30 ± 0.47 (before SO removal) vs. 0.87 ± 0.56 (last follow-up) ** - Improved BCVA = 83.8% - Unchanged in BCVA = 13.5% - Decreased in BCVA = 2.7%.	• IOP > 21 mmHg: 1 eye • Hypopyon: 1 eye Cataract: 2 eyes
Reibaldi M et al. [45] (2013) Italy	Case report	1. PVR development 2. BCVA improvement	1 RRD eye + PVR grade B treated by SB	DEX implant at the end of SB	9 months	1. PVR development: No PVR sign 2. BCVA improvement: from hand-motion to 0.2 LogMAR	No rise of IOP
Mirshahi et al. (2014) [46] Iran	Prospective consecutive case series	1. BCVA improvement 2. CME rate 3. Incidence of persistence SRF 4. Extent of detachment 5. Post-operative inflammation (conjunctival injection)	62 macula off-RRD eyes treated by SB	GROUP 1 (29 EYES) received IVT TA (2 mg) at the end of SB GROUP 2 (33 eyes) received IVT NaCl at the end of SB	1 week, 1, 2, 3 month	1. BCVA improvement: 1. 1 week and 1–2 month group 1 vs. group 2 *** 2. 3 month group 1 > group 2 ** 2. CME rate: group 1 (20,75%) vs. group 2 (33.3%) *** 3. Incidence of persistence SRF: Group 1 (34%) vs. group 2 (45%) *** 4. Extent of detachment: group 1 vs. group 2 *** 5. Post-operative inflammation (conjunctival injection): group 1 (4 eyes-13%) vs. group 2 (10 eyes - 30%) ** There was no correlation between the incidence of persistent SRF and extent of the detachment in both groups (*p* = 0.83)	- Rise of IOP > 21 mmHg in 4 eyes of group 1 No cataract progression
Sherif M and Wolfensberger TJ. [47] (2017) Switzerland	Retrospective review	1. BCVA improvement 2. Stable retinal reattachment with SO 3. Stable retinal reattachment with removal of SO	5 recurrent RRD + PVR stage C + retinal edema	PPV + membrane peeling+ retinectomy+ DEX implant + 5500cs SO	8.8 ± 6.4 months under SO 4–8 months in 3 of the 5 eyes	1. BCVA improvement patient 1: from 0.15 to 0.32 logMAR patient 2: from HM to 0.05 logMAR patient 3: from HM to 0.1 logMAR patient 4: from HM to 0.2 logMAR patient 5: from CF to 0.05 logMAR 2. Stable retinal reattachment with SO: 5 out of 5 eyes 3. Stable retinal reattachment with removal of SO 3 out of 5 eyes	-
Banerjee PJ at al. [48] (2017) United Kingdom	Prospective randomized controlled clinical trial	1. Stable retinal reattachment with removal of SO without additional surgical intervention at 6 months 2. Final VA and proportion of patients achieving a VA of 55 letters 3. Macular findings at 6 months 4. Development PVR recurrence 5. Retinal reattachment: complete and posterior (post-equatorial) 6. Quality of life	140 RRD eyes	GROUP 1 (70 eyes): PPV + SO GROUP 2 (70 eyes): PPV + SO + DEX implant	2 years	1. Stable retinal reattachment with removal of SO without additional surgical intervention at 6 months: 42% (group 1) vs. 49% (group 2) *** 2. Final VA: - ETDRS letters at 6 months: group 1 (40.2 letters - SD = 21.1) vs. group 2 (38.3 letters - SD = 23.7) *** - Proportion of patients achieving a visual acuity of 55 ETDRS letters or better: group 1 (24%) vs. group 2 (30%) *** 3. Macular findings at 6 months: - Macular edema group 1 (67.2%) vs. group 2 (42.7%) ** - Median foveal thickness and macular volume group 1 (365 μ and 9.23 mm^3^) vs. group 2 (297 μ and 8.85 mm^3^) - Proportion of eyes with foveal thickness ≧ 300 μ group 1 (67.7%) vs. group 2 (47.6%) ** 4. Development PVR recurrence: group 1 (59%) vs. group 2 (57%) 5. Retinal reattachment: - Complete group 1 (62.3%) vs. group 2 (53.6%) - Posterior group 1 (69.6%) vs. group 2 (69.7%) 6. Quality of life: no difference using Mean Social Functioning 36-point Questionnaire and Visual Functioning 25-point Questionnaire: no differences between the 2 treatment groups	1. Hypotony (at least 1 episode): group 1 (24.3%) vs. group 2 (20%) 2. IOP (at least 1 episode): group 1 (31.4%) vs. group 2 (45.7%) 3. Macular pucker/epiretinal membranes: group 1 (58.6%) vs. group 2 (57.1%) 4. Patients undergoing cataract surgery: group 1 (86.1%) vs. group 2 (75.8%) 5. Tractional retinal detachment: group 1 (19%) vs. group 2 (22%)
Cho AR Et Al. [49] (2019) Korea	Retrospective interventional case series	1. BCVA 2. Retinal attachment rate and maintenance	7 eyes—AD patients with RD	PPV+ SO + DEX implant	15–37 months	1. BCVA maintained in 5 out of 7 eyes. 2. Retinal attachment rate and maintenance: 100%.	- 1 eye required additional procedure for a recurrent inferior RD at 2 months. - Uveitis: 1 eye. - IOP > 21 mmHg: surgical iridotomy (1 eye), Ahmed valve (2 eyes) topical treatment (3 eyes). - PVR recurrence: 1 eye who had history of multiple PPV - 1 eye repeated DEX implants injection for CME
Reibaldi M et al. [50] (2019) Italy	Prospective randomized multicenter double-blind trial	1. Complete response (absence of PONV: no nausea, no vomiting, no retching, no use of anti-emetic rescue medication) 2. Severity standardized score of PONV (higher intensity) 3. Postoperative pain	1287 eyes affected by various disease treated by PPV GROUP A = 181 RRD eyes GROUP B = 183 RRD eyes GROUP C = 187 RRD eyes GROUP D = 190 RRD eyes	GROUP A (321 eyes) Placebo (IV at the start and 15 min before the end of surgery) GROUP B (316 eyes) ondansetron (4 mg diluted to 10 mL IV 15 min before the end of surgery) + placebo (at the start of surgery) GROUP C (328 eyes) DEX (4 mg diluted to 10 mL IV at the start of surgery) + placebo (15 min before the end of surgery) GROUP D (322 eyes) DEX (4 mg diluted to 10 mL IV at the start of surgery) + ondansetron (4 mg diluted to 10 mL IV 15 min before the end of surgery)	24 h after surgery	1. Complete response (absence of PONV: no nausea, no vomiting, no retching, no use of anti-emetic rescue medication): GROUP D (95.96%, 309/322 patients) vs. GROUP B (80.38%, 254/316 patients), GROUP C (80.79%, 265/328 patients), GROUP A (71.96%, 231/321 patients) ** GROUP B (80.38%, 254/316 patients), GROUP C (80.79%, 265/328 patients) vs. GROUP A (71.96%, 231/321 patients) ** GROUP B (80.38%, 254/316 patients) vs. GROUP C (80.79%, 265/328 patients) *** 2. Severity standardized score of PONV (higher intensity): GROUP A > GROUP B GROUP A > GROUP C GROUP A >> GROUP D ** GROUP B vs. GROUP C *** Postoperative pain ***	No serious non-ocular adverse events CD: GROUP A (4) GROUP B (3) GROUP C (3) GROUP D (2) IOP ≥ 30 mmHg: GROUP A (12) GROUP B (8) GROUP C (9) GROUP D (8) Hypotony ≤ 6 mmHg: GROUP A (4) GROUP B (6) GROUP C (3) GROUP D (7) Suprachoroidal hemorrhage: GROUP A (2) GROUP B (1) GROUP C (1)
				GROUP D (322 eyes) DEX (4 mg diluted to 10 mL IV at the start of surgery) + ondansetron (4 mg diluted to 10 mL IV 15 min before the end of surgery)		3. Postoperative pain ***	RD: GROUP A (1) GROUP C (1) Vitreous hemorrhage: GROUP A (2) GROUP B (2) GROUP C (5) GROUP D (3)

Footnote: ** Statistical significance; *** Non statistical significance difference between the groups; ^ Change from preoperative to 6 months postoperative after silicon oil removal; AC = Anterior chamber; AD = Atopic dermatitis; BCVA = Best corrected visual acuity; CD = choroidal detachment; CME = Cystoid macula edema; DEX implant = 0.7 mg slow release dexamethasone; ERM = Epiretinal membrane; ETDRS = Early treatment diabetic retinopathy study; IOP = Intraocular pressure; IV = intravenous; IVTA = Intravitreal Triamcinolone Acetonide; MH = Macular hole; PDR = Proliferative diabetic retinopathy; PONV = Postoperative nausea and vomiting; PPV = Pars plana vitrectomy; PVR = Proliferative vitreoretinopathy; RD = Retinal detachment; RRD = Rhegmatogenous retinal detachment; RRDCD = Rhehgmatogenous retinal detachment with combined choroidal detachment; SB = Scleral buckling; SD = Standard deviation; SO = Silicone oil; SRF = Sub retinal fluid; TA = Triamcinolone Acetonide; VA = Visual acuity.

**Table 3:** Postoperative use of corticosteroids in retinal detachment surgery.

Author. Year	Study Design	Outcomes	Number of Eyes	Primary Treatment	Follow-Up	Main Results	Side Effects
Dehghan MH et AL. [73] (2009) Iran	Randomized double-blind placebo-controlled trials	1. BCVA logMar 2. Retinal redetachment 3. Macular edema 4. PVR	52 RRD eyes + PVR grade a or B treated by SB	GROUP 1 (25 eyes) post-operative oral prednisolone for 10 days GROUP 2 (27 eyes) placebo	6 months	1. BCVA logMar - Final group 1 (0.62 ± 0.39) vs. group 2 (0.78 ± 0.58) *** - Difference between preoperative and postoperative: group 1 (0.85 ± 0.62) vs. group 2 (0.65 ± 0.61) *** 2. Macular edema within 6 weeks: group 1 (12%) vs. group 2 (18.5) *** 3. PVR within 6 months: group 1 (4%) vs. group 2 (11.1%) ***	CD (within 1 week): group 1 (16%) vs. group 2 (11.1%) ***
Wu JS et al. [74] (2011) Taiwan	Prospective interventional study	1. SRF incidence and duration 2. BCVA (logMar)	60 RRD eyes treated by SB	GROUP 1 (30 eyes) Oral prednisone for 3 days post-SB: - GROUP 1A (11 eyes) = 0.5 mg/kg - GROUP 1B (19 eyes) = 1 mg/kg GROUP 2 (30 eyes) No oral prednisolone post-SB	1 year	1. SRF - Incidence (6 weeks after operation): group 1 (56.6%) vs. group 2 (80%) group 1A (54.5%) vs. group 1B (57.9%) *** - Duration days: group 1 (218.1 ± 122.1 days) vs. group 2 (286.5 ± 141 days) ** group 1A (188.0 ± 114.1) vs. group 1B (237.2 ± 128.5) *** 2. BCVA (logMar) - Final (12 months): group 1 (0.27 ± 0,28) vs. group 2 (0.29 ± 0.31) *** group 1A (0.38 ± 0.36) vs. group 1B (0.20 ± 0.19) *** - BCVA improvement: group 1 (1.38 ± 1.05) vs. group 2 (0.74 ± 0.78) ** group 1A (1.47 ± 0.94) vs. group 1B (1.22 ± 1.24) ***	No systemic complications
Koemer F et al. [75] (2012) Switzerland	Prospective randomized, placebo, controlled, double blind clinical trial	1. PVR stage B incidence 2. Cellophane appearance 3. Retinal rigidity 4. Epiretinal membranes 5. PVR stage C	220 RRD eyes treated by SB	GROUP 1 (110 eyes) Oral prednisolone after SB GROUP 2 (110 eyes) Placebo after SB	30, 90, 180 days	1. PVR stage B incidence: - 30 days group 1 (26.4%) vs. group 2 (40.4%) ** - 90 days group 1 (25.2%) vs. group 2 (45.5%) ** - 180 days group 1 (22.5%) vs. group 2 (45.7) ** 2. Cellophane appearance: group 1 vs. group 2 *** 3. Retinal rigidity: group 1 vs. group 2 *** 4. Epiretinal membranes: group 1 vs. group 2 *** 5. PVR grade C: group 1 vs. group 2 ***	—
Ben YS et al. [7] (2016) Tunisia	Prospective study	1. Intensity of post-operative pain by VAS 2. ACAF 3. IOP	40 RRD eyes	GROUP 1 (28 eyes) treated by SB: - GROUP 1A = 13 eyes (46.4%) topical dexamethasone 4 times daily for 28 days after surgery - GROUP 1B = 15 eyes (53.6%) topical diclofenac sodium 0,1% 3 times daily for 28 days after surgery GROUP 2 (12 eyes) treated by PPV: - GROUP 2A = 6 eyes (50%) dexamethasone - GROUP 2B = 6 eyes (50%) diclofenac	7. 14. 28. 90 (days)	1. Intensity of post-operative pain: GROUP1 - 7 days = group 1A (2.48 ± 0.94) vs. group 1B (1.77 ±0,87) ** - 14 days = group 1A (1.06 ± 0.45) vs. group 1B (0.43 ± 0.63) ** - 28 days = group 1A (0.5 ± 0.35) vs. group 1B (0.26 ± 0.18) ** - 90 days = group 1A vs. group 1B *** GROUP 2 - 7 days = group 2A (1.92 ± 0.87) vs. group 2B (1.73 ± 0.78) ** - 14 days = group 2A (0.82 ± 0.39) vs. group 2B (0.28 ± 0.34) ** - 28 days = group 2A (0.4 ± 0.19) vs. group 2B (0.14 ± 1.14) ** - 90 days = group 2A vs. group 2B *** 2. ACAF (ph/ms): group 1A vs. group 1B *** group 2A vs. group 2B *** 3. IOP: group 1A vs. group 1B *** group 2A vs. group 2B ***	No post-operative complications
Yasuda K et al. [76] (2016) Japan	Prospective interventional study	1. ACAF 2. IOP 3. BCVA 4. Total OT 5. Dynamic maxPOI changes 6. maxPOI	200 eyes with CATARACT + MH/ERM/DME/RRD + SF6	Sutureless cataract surgery + PPV: - GROUP 1: 106 eyes treated by topic post-operative diclofenac 0.1% (18 RRD) - GROUP 2: 85 topic post-operative betamethasone 0.1% (18 RRD)	12 weeks	1. ACAF: Group 1 = RRD (10.3 ± 3.5) vs. other disease *** Group 2 = RRD (11.9 ± 6.4) vs. other disease *** Group 1 vs. Group 2 *** 2. Pre-operative IOP: Group 1 = RRD (12.6 ± 2.8) < other disease ** Group 2 = RRD (12.6 ± 4.2) < other disease ** Post-operative IOP: RRD = Group 2 > Group 1 ** 3. BCVA: Group 1 vs. Group 2 *** 4. Total OT: Group 1 = RRD (40.9 ± 6.8) > other disease ** Group 2 = RRD (44.1 ± 12.4) > other disease ** Group 1 vs. Group 2 *** 5. Dinamic maxPOI changes:Group 1 vs. Group 2 *** 6. RRD maxPOI: significantly correlated with the number of endophotocoagulations, total OT in group 1 and indentation during PPV in group 1 and 2	—

Footnote: ** statistical significance; *** non statistical significance difference between the groups; ACAF = Anterior chamber aqueous flare; BCVA = Best corrected visual acuity; CD = Choroid detachment; DME = Diabetic macular edema; ERM = Epiretinal membrane; IOP = Intraocular pressure; MH = Macular hole; Max POI= Maximum postoperative inflammation index (maxPOI); PPV = Pars plana vitrectomy; PVR = Proliferative vitreoretinopathy; RRD = Rhegmatogenous retinal detachment; SB = Scleral buckling; SRF = Sub retinal fluid; VAS = Scott’s visual analog scale consists of a 10-cm scale along with a cursor moved by the patient along a straight line, one end corresponds to “no pain” and the other end corresponds to “maximum imaginable pain.”
